# A Comparison of HAART Outcomes between the US Military HIV Natural History Study (NHS) and HIV Atlanta Veterans Affairs Cohort Study (HAVACS)

**DOI:** 10.1371/journal.pone.0062273

**Published:** 2013-05-01

**Authors:** Jodie L. Guest, Amy C. Weintrob, David Rimland, Christopher Rentsch, William P. Bradley, Brian K. Agan, Vincent C. Marconi, IDCRPHIV Working Group

**Affiliations:** 1 Atlanta VA Medical Center, Atlanta, Georgia, United States of America; 2 Emory University School of Medicine, Atlanta, Georgia, United States of America; 3 Rollins School of Public Health at Emory University, Atlanta, Georgia, United States of America; 4 Infectious Disease Clinical Research Program, Uniformed Services University of the Health Sciences, Bethesda, Maryland, United States of America; 5 Walter Reed National Military Medical Center, Bethesda, Maryland, United States of America; Mayo Clinic, United States of America

## Abstract

**Introduction:**

The Department of Defense (DoD) and the Department of Veterans Affairs (VA) provide comprehensive HIV treatment and care to their beneficiaries with open access and few costs to the patient. Individuals who receive HIV care in the VA have higher rates of substance abuse, homelessness and unemployment than individuals who receive HIV care in the DoD. A comparison between individuals receiving HIV treatment and care from the DoD and the VA provides an opportunity to explore the impact of individual-level characteristics on clinical outcomes within two healthcare systems that are optimized for clinic retention and medication adherence.

**Methods:**

Data were collected on 1065 patients from the HIV Atlanta VA Cohort Study (HAVACS) and 1199 patients from the US Military HIV Natural History Study (NHS). Patients were eligible if they had an HIV diagnosis and began HAART between January 1, 1996 and June 30, 2010. The analysis examined the survival from HAART initiation to all-cause mortality or an AIDS event.

**Results:**

Although there was substantial between-cohort heterogeneity and the 12-year survival of participants in NHS was significantly higher than in HAVACS in crude analyses, this survival disparity was reduced from 21.5% to 1.6% (mortality only) and 26.8% to 4.1% (combined mortality or AIDS) when controlling for clinical and demographic variables.

**Conclusion:**

We assessed the clinical outcomes for individuals with HIV from two very similar government-sponsored healthcare systems that reduced or eliminated many barriers associated with accessing treatment and care. After controlling for clinical and demographic variables, both 12-year survival and AIDS-free survival rates were similar for the two study cohorts who have open access to care and medication despite dramatic differences in socioeconomic and behavioral characteristics.

## Introduction

In the HAART era, individuals with HIV are living longer after their HIV diagnoses [1]. When compared to similar populations diagnosed with HIV prior to HAART availability, individuals with HIV in the modern HAART era suffer a range of health challenges that are associated with aging and therapy, including comorbid illnesses [2]. The complex nature of the treatment for these diseases can be a significant barrier for patients attempting to remain adherent with care. The same risk factors associated with HIV transmission such as poverty, discrimination, and inadequate education, persist or become exacerbated after HIV infection and can also serve as major barriers to healthcare retention [3], . Other barriers are inherent to the healthcare system and payer source, such as enrollment applications, pre-existing conditions and copayments. When compounded with the barriers that are specific to the individual, these impediments can result in treatment discontinuation and adverse clinical outcomes which can ultimately impact on the overall cost-effectiveness of HIV treatment and care for a particular program. Thus, complex and chronic diseases require an integration of multiple services and providers and as few socioeconomic obstacles as possible in order to simultaneously improve outcomes and increase efficiency [5], [6]. It has not been clearly demonstrated how a healthcare system should be organized to address these challenges within a defined amount of resources.

Providing integrated, multi-disciplinary healthcare for individuals with HIV has been a significant challenge for most clinicians functioning within the constraints of a contemporary practice. In addition to primary care services, such a system usually requires a broad range of medical services, including a full-service pharmacy, a laboratory, comprehensive prevention and education programs, mental health services, and physical therapy. Both the Department of Defense (DoD) and the Department of Veterans Affairs (VA) provide comprehensive HIV treatment and care to their beneficiaries with little or no out-of-pocket costs to the patient and with open access.

In preparation for subsequent cost effectiveness comparisons, this study aimed to compare clinical outcomes associated with HIV treatment and care for patients from both the HIV Atlanta VA Cohort Study (HAVACS) and the US Military HIV Natural History Study (NHS) cohorts. The Department of Defense (DoD) and the Department of Veterans Affairs (VA) provide comprehensive HIV treatment and care to their beneficiaries with open access and few costs to the patient. This comparison between individuals receiving HIV treatment and care from the DoD and the VA provided an opportunity to explore the impact of individual-level characteristics on clinical outcomes within two healthcare systems that are optimized for clinic retention and medication adherence. Furthermore, we sought to characterize, when possible, the relative contribution of these individual-level factors to clinical outcomes when access to care is not a confounding issue.

## Methods

### Study Participants

Data were collected from the two cohorts: the HAVACS and the NHS. The NHS has enrolled over 5000 beneficiaries into a prospective, multicenter observational study of active duty military personnel and other military beneficiaries living with HIV since 1986. The NHS cohort characteristics have been previously described [7]. As a result of routine HIV screening in the military, NHS cohort members are diagnosed early and the majority has estimated dates of seroconversion. The HAVACS includes all veterans living with HIV who have received treatment and care at the Atlanta VA Medical Center since 1982 (n>3800). The cohort characteristics of HAVACS have been previously described [8]. Data have been prospectively collected for both cohorts and are used for clinical care and research purposes. The NHS cohort has been approved by the Institutional Review Board (IRB) centrally (Uniformed Services University of the Health Sciences, Bethesda, MD) and at each participating center (Walter Reed National Military Medical Center, Bethesda, MD; Naval Medical Center, Portsmouth, VA; San Antonio Military Medical Center, TX; Naval Medical Center, San Diego, CA; and Tripler Army Medical Center, Honolulu, HI). Written consent was obtained from each patient. The HAVACS cohort has been approved by Emory University's IRB and the Atlanta VA Medical Center Research and Development Committee. The HAVACS cohort does not require patients' written consent as it has an IRB-approved HIPAA waiver.

Patients in both cohorts were eligible for inclusion in this analysis if they had an HIV diagnosis and began HAART between January 1, 1996 and June 30, 2010. A total of 1199 NHS patients and 1065 HAVACS patients were followed from their recorded HAART initiation date through all-cause mortality, an AIDS-defining event, or the date of last data entry for these analyses.

### Variable Definitions

For the survival analyses, an AIDS-defining event utilized the 1993 CDC definition of AIDS; however, given the amount of HAVACS patients who had CD4 counts below 200 cells/mm^3^ at study enrollment, CD4<200 was not included in this definition but was analyzed separately. A HAART regimen was defined as the use of three or more antiretroviral medications, one of which has to be a protease inhibitor (PI), a non-nucleoside reverse transcriptase inhibitor (NNRTI), an integrase inhibitor, or an entry inhibitor. A participant's HAART initiation date was the date of the first HAART regimen that lasted greater than one month. This regimen was recorded as the participant's initial HAART regimen, regardless of any subsequent changes in drugs, thus utilizing an intention to treat format for this variable. Information regarding the participant's age at HAART initiation, sex, race, year of HIV diagnosis, viral load and CD4 count at HAART initiation (within three months of HAART start date), history of chronic hepatitis B and C co-infection, and previous ARV use (mono or dual NRTI) were also analyzed.

**Figure 1:**
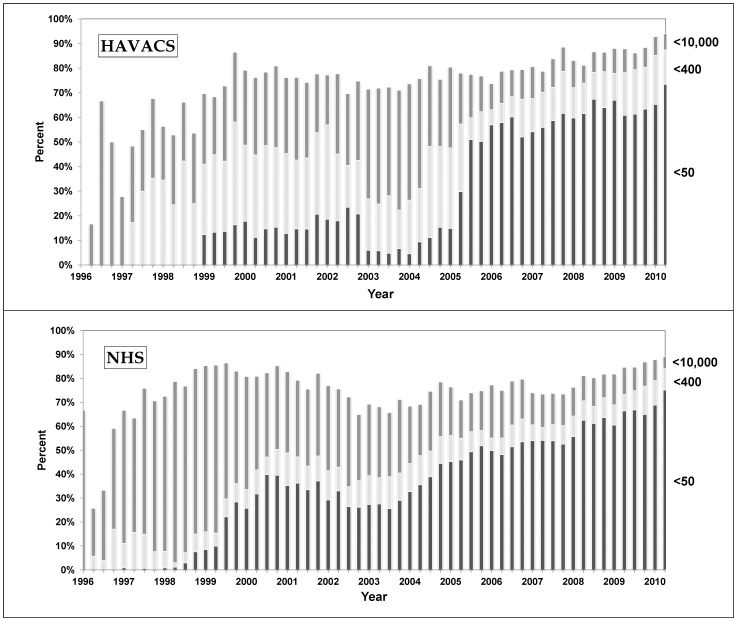
Quarterly HIV viral load distributions within each HAART-initiating cohort from January 1, 1996 to June 30, 2010.

The distribution of HIV-1 viral loads was determined by the values available in each three month period from 1996 through 2010. These were then presented as values <50, 51–400, 400 to 10,000, and greater than 10,000 copies/ml (Figure 1).

### Statistical Analysis

Potential variables were selected based on previously published findings regarding influences on survival of patients living with HIV in the presence of HAART [9]. Two-sided Wilcoxon rank sum and chi-square (χ^2^) tests were used to compare the selected variables across the two cohorts. The variables were subsequently screened using bivariate analyses and were dropped from further inclusion in multivariate models if their crude association's *p*-value with the outcomes were greater than 0.5. Only one variable, year of HIV diagnosis, did not meet the threshold for inclusion into the multivariate analyses. All interactions were considered and dropped using a backwards elimination approach with a likelihood ratio test for significance. All remaining variables were assessed for collinearity. Variables that remained after these procedures were utilized throughout all analyses for comparison between models. The proportional hazards (PH) assumptions were gauged for each variable utilizing a consensus of three different approaches: graphical, goodness of fit, and extended Cox modeling. All variables of interest satisfied the PH assumption. The Akaike Information Criterion (AIC) was also used to determine if a credible difference between a full model using all variables of interest and a more parsimonious model using any combination of variables might exist [10]. The full model's AIC value (AIC = 1778.4) was similar to the parsimonious model's AIC value (AIC = 1773.6); thus, the full model was chosen and these results were reported.

Two separate outcomes were assessed for each cohort: time to all-cause mortality and time to all-cause mortality or an AIDS-defining event (excluding CD4<200), whichever occurred first. For both, follow-up began at time of HAART initiation. Cox proportional hazards models were fitted to investigate mortality and AIDS rates by cohort. Survival curves, both unadjusted and adjusted, were estimated using the corrected group prognosis method [11]. Analyses were conducted with SAS 9.2 (SAS, Cary, NC, USA).

## Results

### Baseline and Demographic Characteristics

Table 1 shows the substantial between-cohort heterogeneity. HAVACS participants were older at HAART initiation with a median age of 42 years compared to 31 years in NHS (*p*<0.0001). Although both cohorts are predominantly male (<5% of the total study population was female), the NHS cohort did have significantly more females than did HAVACS (*p*<0.0001). Eighty percent of HAVACS patients were African-American/Black while the NHS cohort was more evenly divided between African-American/Black and European-American/White race (42.6% and 41.0%, respectively) (*p*<0.0001). Given the lack of other races represented in HAVACS, no other race was specifically analyzed although 16.4% of the NHS was neither African-American/Black nor European- American/White.

**Table 1:** Distributions of variables between NHS and HAVACS.

Variable	Cohort		
	NHS (n = 1199)	HAVACS (n = 1065)	P^a^
**Demographics**			
Age at HAART initiation, years^b^	31.0 (25.0–37.0)	42.0 (35.0–51.0)	<0.0001
Female	6.8% (82)	2.5% (27)	<0.0001
Race			
*African-American/Black*	42.6% (511)	78.8% (838)	<0.0001
*European-American/White*	41.0% (491)	19.8% (211)	
*Other*	16.4% (197)	1.4% (15)	
**Medical history (prior to HAART initiation)**			
Year of HIV diagnosis	2001±4.0	2000±3.8	0.0003
HIV diagnosis to HAART initiation, months^b^	4.7 (1.6–16.9)	5.0 (1.2–23.3)	0.6774
Viral load at HAART initiation, log copies/mL^b^	4.6 (4.0–5.0)	4.8 (4.2–5.4)	<0.0001
*<2.60*	5.9% (67)	6.6% (44)	0.0138
*2.60*–*4.00*	17.6% (201)	12.5% (83)	
*>4.00*	76.5% (872)	80.9% (539)	
Nadir CD4+ to HAART initiation, months^b^	0.9 (0.2–3.0)	1.0 (0.8–3.9)	<0.0001
CD4+ at HAART initiation, cells/mm^3^ b	341.0 (251.0–461.0)	188.0 (49.5–296.5)	<0.0001
*<200*	14.1% (159)	53.0% (443)	<0.0001
*200*–*349*	37.4% (420)	28.3% (237)	
*350+*	48.5% (545)	18.7% (156)	
Previous AIDS-defining event^c^			
*AIDS-defining event (inclusive of CD4)*	16.6% (199)	54.8% (584)	<0.0001
*AIDS-defining event (exclusive of CD4)*	3.4% (41)	20.2% (215)	<0.0001
*CD4<200/14%*	15.1% (181)	39.3% (419)	<0.0001
Chronic hepatitis B co-infection	1.5% (18)	5.5% (59)	<0.0001
Hepatitis C co-infection	2.8% (33)	12.7% (135)	<0.0001
Previous ARV use (mono or dual therapy)	16.1% (193)	19.7% (210)	0.0245
**Initial HAART regimen**			
Unboosted PI	27.7% (333)	28.4% (302)	<0.0001
Boosted PI	13.0% (154)	16.0% (170)	
NNRTI	51.3% (610)	52.6% (560)	
PI/NNRTI/NRTI	2.0% (24)	0.3% (3)	
3NRTI	6.0% (71)	2.8% (30)	
**Outcomes**			
Died	3.2% (38)	17.1% (182)	<0.0001
AIDS after HAART initiation^c^			
*AIDS-defining event (inclusive of CD4)*	6.4% (77)	24.0% (256)	<0.0001
*AIDS-defining event (exclusive of CD4)*	2.6% (31)	13.2% (140)	<0.0001
*CD4<200/14%*	4.4% (53)	12.9% (137)	<0.0001

NHS-US Military HIV Natural History Study; HAVACS-HIV Atlanta VA Cohort Study; HAART-Highly Active Antiretroviral Therapy; IQR-interquartile range; PI-Protease Inhibitor; NNRTI-Non-Nucleoside Reverse Transcriptase Inhibitor; NRTI-Nucleoside Reverse Transcriptase Inhibitor.

a Tested for significance with two-sided Wilcoxon rank-sum and chi-square (χ^2^) tests.

b Showed in median units (IQR).

c AIDS-defining event utilizes the 1993 CDC definition.

### Clinical, Virologic and Immunologic Characteristics

HAVACS participants were more likely to have had an AIDS-defining event (whether including or excluding CD4<200) prior to HAART initiation and were more likely to be hepatitis C and hepatitis B co-infected at HAART initiation (all *p*<0.0001). HAVACS patients had a lower CD4 cell count at HAART initiation with a median count of 188.0 cells/mm^3^ (IQR 49.5–296.5) compared to 341.0 cells/mm^3^ (IQR 251.0–461.0) at HAART initiation for patients in the NHS (*p*<0.0001). The distribution of viral load measurements at HAART initiation were less striking between the cohorts but were still significantly different with HAVACS participants having higher viral load levels (median 4.8 log copies/mL; IQR 4.2–5.4) compared to NHS participants (mean 4.6 log copies/mL; IQR 4.0–5.0) (*p*<0.0001).

### Antiretroviral Usage

Although the HAVACS participants were diagnosed with HIV in an earlier calendar year than those in the NHS, there was no statistically significant difference in time from HIV diagnosis to HAART initiation (approximately five months), although there was substantial variability in this time. Patients in HAVACS were more likely to have used mono or dual ARV therapy before HAART (19.7% vs. 16.6%, *p* = 0.0245); however, based on year of HIV diagnosis and years included in this analysis, this was not a common event. Both cohorts had an average time to HAART initiation from nadir CD4 of one month or less, yet the difference between these cohorts was still statistically significant (*p*<0.0001). Although the type of initial HAART regimen was statistically significantly different between the two cohorts, both groups were most likely to initiate HAART with a NNRTI-containing regimen and less likely to have initiated HAART with either a regimen containing a PI or three NRTIs.

### Outcomes

During 185,356 person-months of follow-up, 220 (9.7%) patients died. The majority of these patients were from HAVACS (n = 182, 17.1%) compared with the NHS (n = 38, 3.2%) (*p*<0.0001). HAVACS patients were also more likely to have an AIDS-defining event after HAART initiation; 13.2% of HAVACS patients compared to 2.6% of NHS patients (*p*<0.0001). When CD4<200 cells/mm^3^ was included, 24.0% of HAVACS patients had an AIDS-defining event compared to 6.4% of NHS (*p*<0.0001).

The distribution of HIV-1 viral loads by quarter is shown in Figure 1. Before 2005, some differences may have been due to technical issues with processing specimens [12] in HAVACS. After 2005, the distribution of values is very similar, showing that more than 70% of the patients in both cohorts had undetectable viral loads below 50 copies/mL by the end of the study period.

**Figure 2:**
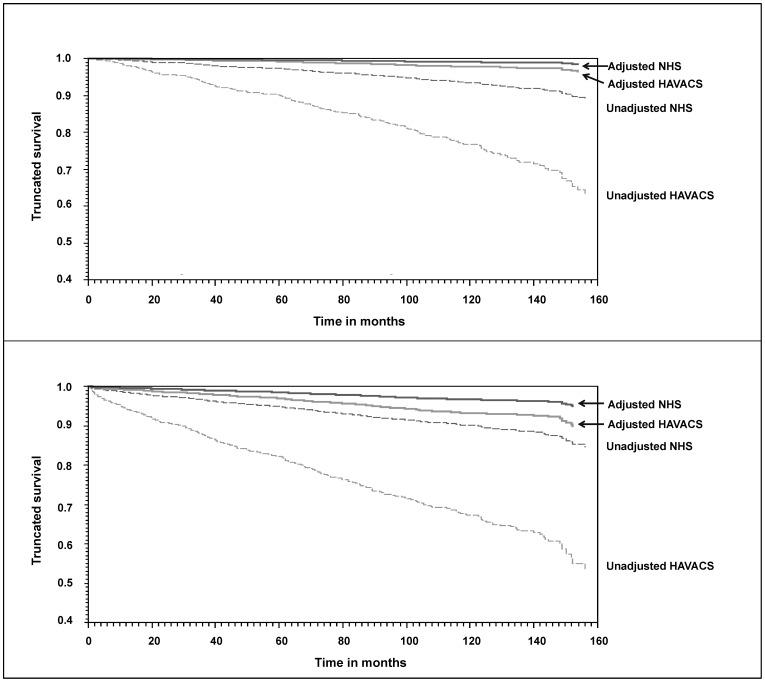
**a.** Unadjusted and adjusted survival curves from HAART initiation to death by cohort. **b.** Unadjusted and adjusted survival curves from HAART initiation to death or AIDS (excl. CD4<200) by cohort.

Table 2 displays the crude hazard ratios (cHR) and adjusted hazard ratios (aHR) for Cox proportional hazards modeling for both outcomes of interest. In all models, the survival of participants in the NHS cohort was significantly greater than in the HAVACS. After controlling for age, sex, race, CD4 and VL at HAART initiation, prior AIDS, Hepatitis B and C, prior ARV usage and regimen, the 12-year survival rates between the cohorts decreased from a 21.5% crude difference (NHS–91.5%; HAVACS–70.0%) to a 1.6% adjusted difference (NHS–98.7%; HAVACS–97.1%) (Figure 2). Similarly, the difference in the 12-year survival rates to combined mortality/AIDS events between the cohorts decreased from a 26.8% crude difference (NHS–87.8%; HAVACS–61.0%) to a 4.1% adjusted difference (NHS–95.8%; HAVACS–91.7%). Patients in the NHS were 57% less likely to die during follow-up compared to patients in HAVACS (aHR 0.43, 95% confidence interval (CI) 0.27, 0.70) and were 51% less likely to have reached the composite outcome of death or AIDS-defining event (aHR 0.49, 95% CI 0.34, 0.71) compared to patients in HAVACS.

**Table 2:** Crude and adjusted predictors of time to development of outcomes after initiating HAART using Cox proportional hazards modeling.

Variable	Death (n = 1727)		Death or AIDS-defining event^a^ (n = 1726)	
	cHR	aHR	cHR	aHR
Cohort, NHS vs. HAVACS	**0.26 (0.18, 0.36)**	**0.43 (0.27, 0.70)**	**0.27 (0.20, 0.35)**	**0.49 (0.34, 0.71)**
**Demographics**				
Age at HAART initiation	**1.07 (1.06, 1.08)**	**1.06 (1.04, 1.08)**	**1.06 (1.05, 1.07)**	**1.04 (1.03, 1.05)**
Sex, female vs. male	2.31 (0.95, 5.61)	2.64 (0.64, 10.80)	**2.54 (1.20, 5.36)**	2.11 (0.78, 5.73)
Race, AA vs. other	**1.63 (1.21, 2.20)**	1.20 (0.80, 1.79)	**1.66 (1.31, 2.12)**	1.08 (0.78, 1.50)
**Medical history (prior to HAART initiation)**				
HIV diagnosis to HAART initiation, years	1.07 (0.98, 1.16)	0.97 (0.87, 1.07)	1.06 (1.00, 1.13)	1.00 (0.92, 1.07)
Viral load at HAART initiation, log copies/mL				
<2.60	0.52 (0.23, 1.17)	0.46 (0.19, 1.15)	**0.41 (0.19, 0.86)**	**0.37 (0.16, 0.83)**
2.60–4.00	1.05 (0.69, 1.60)	1.46 (0.91, 2.33)	0.86 (0.59, 1.26)	1.22 (0.81, 1.85)
>4.00	1.00	1.00	1.00	1.00
CD4+ at HAART initiation, cells/mm^3^				
<200	**3.45 (2.36, 5.04)**	1.28 (0.79, 2.07)	**4.03 (2.94, 5.52)**	**1.62 (1.08, 2.43)**
200–349	1.08 (0.66, 1.76)	0.60 (0.34, 1.03)	1.24 (0.83, 1.83)	0.85 (0.55, 1.32)
350+	1.00	1.00	1.00	1.00
Previous AIDS-defining event^a^	**2.24 (1.63, 3.06)**	0.95 (0.62, 1.43)	**2.70 (2.10, 3.47)**	1.27 (0.90, 1.78)
Chronic hepatitis B co-infection	**2.51 (1.53, 4.12)**	**1.89 (1.10, 3.25)**	**1.94 (1.23, 3.05)**	1.39 (0.84, 2.29)
Hepatitis C co-infection	**3.66 (2.66, 5.03)**	**1.60 (1.08, 2.38)**	**2.76 (2.08, 3.66)**	1.40 (0.99, 1.98)
Previous ARV use (mono or dual therapy)	1.34 (0.99, 1.82)	1.40 (0.95, 2.07)	**1.40 (1.09, 1.80)**	**1.39 (1.00, 1.91)**
**Initial HAART regimen**				
NNRTI	1.00	1.00	1.00	1.00
Boosted PI	1.47 (0.98, 2.22)	**1.78 (1.10, 2.86)**	**1.83 (1.35, 2.50)**	**2.10 (1.44, 3.05)**
Unboosted PI	1.04 (0.76, 1.41)	1.28 (0.86, 1.90)	1.07 (0.83, 1.38)	1.32 (0.95, 1.83)
Other	0.34 (0.14, 0.83)	0.72 (0.25, 2.01)	**0.32 (0.15, 0.69)**	0.45 (0.17, 1.27)

Note: Bold hazard ratios (HR) and 95% confidence intervals (CI) are significant at the *p*<0.05 level.

HAART-Highly Active Antiretroviral Therapy; NHS-US Military HIV Natural History Study; HAVACS-HIV Atlanta VA Cohort Study; AA-African -American/Black; PI-Protease Inhibitor; NNRTI-Non-Nucleoside Reverse Transcriptase Inhibitor.

a 1993 definition, exclusive of CD4<200.

## Discussion

In this analysis, we assessed the clinical outcomes for individuals with HIV from two very similar government-sponsored healthcare systems that were designed to reduce or eliminate many of the barriers associated with accessing treatment and care. Patients with DoD or VA health benefits do not face interruptions in care or eligibility exclusions due to interstate migration, changes in employment or income-level, or pre-existing medical conditions. With few exceptions, medications and care require little or no out-of-pocket fees or copayments for eligible individuals. With these significant barriers removed, even individuals who are homeless, unemployed and suffer from multiple comorbidities and substance abuse disorders can receive high quality, uninterrupted treatment and care. Both populations also share a common origin of active duty service which provides a unique opportunity to examine the impact of changing socioeconomic and behavioral structures in light of similar occupational skills, educational backgrounds and training experiences. However from the data presented, it is clear that the VA population studied differed substantially in nearly all clinical characteristics measured compared to the DoD population studied. The VA population has significantly greater comorbidities such as hepatitis C (also a marker for intravenous drug use, which is nearly absent in the NHS [13]) and hepatitis B co-infection as well as more AIDS-defining events. They are also older and have lower CD4 counts on average than those in the DoD in part reflecting a later adoption of routine HIV screening. By law, routine opt-out testing could not be performed in the VA until August 2009. This is perhaps the most important distinction between these two populations. It is likely that the longer time from HIV seroconversion to HIV diagnosis and subsequent HAART initiation for patients in the VA accounts for most of the clinical differences observed between these two cohorts.

Similarly, previous reports have shown that these two populations also differ substantially with regards to socioeconomic and behavioral characteristics as well as non-AIDS-related comorbidities such as diabetes mellitus (DM), cardiovascular disease (CVD), and pulmonary disease. Though these data were not available on individual patients and thus could not be controlled for in the analyses, the possible impact can be inferred by aggregate numbers illustrating the differences between these two groups. National data from the VA Cohort Study (VACS) show rates of unemployment to be 11–13%, 12% of veterans are currently homeless and 32% have been homeless at some point, 30% suffer from depression, and substance abuse disorders range from 5–63% in this population [14]. In contrast, the DoD housing, employment/income, depression and substance abuse rates from the 2008 DoD Health Related Behaviors survey were respectively 6.2% (difficulty with housing), 8.6% (difficulty with money), 2.3% (any substance abuse excluding prescription drugs), and 21% (need for depression evaluation in past 7 days) [13]. The impact of differences in non-AIDS related comorbidities can also be extrapolated from previously published data. Data from VACS show rates of 8% for DM and 6% for CVD [15], while data for military members on active duty show rates of 4.5% for DM and 5.9% for CVD [16]. Though the data for active duty includes HIV+ and HIV- military members and therefore is not completely comparable, these are the best data available.

Despite large clinical and social differences, the study period survival rates for mortality and combined mortality/AIDS events were dramatically mitigated (19.9% and 22.7% reduction in between-cohort differences, respectively) when adjusting for important clinical and demographic variables. The adjusted survival differences were only 1.6% and 4.1% for each respective endpoint after controlling for these variables. Though both systems provide free or low-cost access to health care for eligible patients with HIV, only the DoD routinely screens for HIV and thus, these patients were generally diagnosed at an earlier stage in their HIV disease. Although patients in both the NHS and HAVACS were likely to be started on HAART within one month of their HIV diagnosis, 48.5% of the NHS patients had a CD4>350 at time of HAART initiation compared to 18.7% of HAVACS patients implying diagnosis at an earlier stage of disease for NHS patients. The impact of early diagnosis cannot be underestimated. Patients who started HAART with a CD4<200 cells/mL were 62.1% more likely to die or have an AIDS-defining event during follow-up (95% CI 1.08, 2.43) when compared to those who started with a CD4>500. Given the DHHS guidelines to start HAART at a CD4>500 cells/mL [17], this is not a surprising finding and continues to stress the need for earlier detection, even in health care systems with no barriers to testing or treatment. It is conceivable that healthcare systems which invest more in both HIV screening and early treatment can ultimately show a cost-savings at the institution and societal levels by achieving stable and superior clinical effectiveness. These upfront costs would involve early and aggressive identification of individuals with HIV infection followed by active participation in a comprehensive, integrated treatment and care program (including disease prevention and wellness education) which has reduced or eliminated many of the system-associated barriers to care [5], [6]. Subsequently, patients would be less likely to experience adverse events related to HIV infection, medications or comorbidities and would require less hospital admissions and salvage treatment that, in turn, would improve quality of life, reduce overall costs and increase worker productivity [18]–[21]. Sustained virologic suppression will also result in fewer secondary HIV transmission events which in turn will result in additional individual, institutional and societal benefits [22].

Our findings are limited by the inability to specifically adjust for socioeconomic and behavioral variables and non-AIDS defining comorbidities which most certainly have an impact on clinical outcomes. Additionally, because the HAVACS patients have a lower CD4 at HAART initiation, this may add some survivor bias to the analysis. Furthermore, since data for a fee-for-care group are unavailable, it is difficult to speculate how these open access to care and medication systems would compare with regard to clinical outcomes.

## Conclusions

We assessed the clinical outcomes for individuals with HIV from two very similar government-sponsored healthcare systems that reduced or eliminated many barriers associated with accessing treatment and care. The combined total mortality rate was less than 10% for those diagnosed with HIV and initiated HAART since 1996. The NHS patients were younger and started HAART sooner during HIV infection with a higher CD4 cell count and experienced less mortality than HAVACS patients. Despite these substantial differences in baseline characteristics and large differences in crude mortality rates, after controlling for important clinical and demographic variables, both 12-year survival and AIDS-free survival rates were similar between the two study cohorts who have open access to care and medication. These similarities existed despite dramatic differences in socioeconomic and behavioral characteristics. The remaining significant difference in survival may be due to uncontrolled social issues, and additional non-AIDS comorbidities (such as DM or CVD). Future studies should investigate comparisons of clinical outcomes in fee-for-care systems and factors including drug abuse, mental health issues, and socioeconomic differences, as well as impact of early HIV screening. Our data suggest that early screening, with subsequent higher CD4 at diagnosis, will result in improved outcomes.
